# Effect of Incorporation of Mg on LiTa_0.6_Nb_0.4_O_3_ Photocatalytic Performance in Air-Cathode MFCs for Bioenergy Production and Wastewater Treatment

**DOI:** 10.3390/nano15241837

**Published:** 2025-12-05

**Authors:** Fouzia Allali, Kaoutar Kara, Siham Elmazouzi, Noureddine Lazar, Latifa Tajounte, Noureddine Touach, Abdellah Benzaouak, El Mostapha Lotfi, Abdelilah Lahmar, Leonarda Francesca Liotta

**Affiliations:** 1 Laboratory of Spectroscopy, Molecular Modelling, Materials, Nanomaterials, Water and Environment, Environmental Materials Team, École Nationale Supérieure d’Arts et Métiers (ENSAM), Mohammed V University in Rabat, B.P. 6207 Avenue des Forces Armées Royales, Rabat P.O. Box 1014, Morocco; allali.fouzia88@gmail.com (F.A.); k.kara@um5r.ac.ma (K.K.); noureddinelazar@yahoo.fr (N.L.); l.tajounte@um5r.ac.ma (L.T.); n.touach@um5r.ac.ma (N.T.); el-mostapha.lotfi@ensam.um5.ac.ma (E.M.L.); 2 Physical Chemistry of Materials Laboratory, Ben M’Sick Faculty of Sciences, Hassan II University, Boulevard Cdt Driss El Harti, Casablanca P.O. Box 7955, Morocco; siham.elmazouzi16@gmail.com; 3 Laboratoire de Physique de la Matière Condensée (LPMC), Université de Picardie Jules Verne, Pôle Scientifique, CEDEX 1, 80039 Amiens, France; abdel.ilah.lahmar@u-picardie.fr; 4 Istituto per lo Studio dei Materiali Nanostrutturati (ISMN)-CNR, Via Ugo La Malfa, 153, 90146 Palermo, Italy

**Keywords:** ferroelectric, power density, waste water, COD, electrochemical, voltammetry, physico-chemical, catalyst

## Abstract

Microbial fuel cells are a new alternative for sustainable energy generation and wastewater treatment technology. To scale up this technology, cost-effective electrodes are required. The electrochemical reduction of oxygen at the cathode is a key reaction for power generation. Noble metals, especially Pt, are extensively used as cathode catalysts in MFC; however, its application is limited to its high cost and catalyst poisoning. Ferroelectric materials are reported as a good candidate due to their spontaneous polarization. The main objective of this study is to prepare and characterize the cost-effective ferroelectric materials LiTa_0.6_ Nb_0.4_ O_3_ and Li_0.95_ Ta_0.57_ Nb_0.38_ Mg_0.15_ O_3_ in order to test their catalytic activity in air-cathode MFC. Powders were prepared following the solid-state synthesis and characterized using Scanning Electron Microscopy, energy-dispersive X-ray spectroscopy, and X-ray diffraction. To evaluate the electrochemical performance of the catalysts, electrochemical studies such as EIS, CV, LSV, and CA were conducted. In MFC, the performance of our material has been investigated using COD determination and polarization measurement. The obtained results demonstrate the potential of Li_0.95_ Ta_0.57_ Nb_0.38_ Mg_0.15_ O_3_ as a low-cost and effective catalyst material in MFCs, showing a high COD removal up to 75%, and power-density output of 764 mW/m^2^.

## 1. Introduction

Industry development and human population growth lead to excessive energy demand and increased environmental problems, which necessitates the development of renewable and sustainable energy sources [1]. Currently, microbial fuel cells (MFCs) have been widely studied as a promising solution, using microbes to generate electrical energy while removing pollutants [2,3]. Systematic MFCs consist of an anode chamber and a cathode chamber; in the anode compartment, the organic matter contained in waste water is metabolized to produce electrons and protons (oxidation reaction), and the produced electrons are transported to the cathodic compartment through an external circuit. However, protons are transferred via a PEM, generally used as a separator in typical MFCs. The reduction reaction with oxygen occurs in the cathode [4].

The performance of MFCs depends on several factors, such as the material of the electrode at the anode and cathode, the nature of treated wastewater, the type of separator used, the configuration of the MFC, and the operational conditions (temperature, pH, …) [5,6].

The overall power output efficiency of an MFC is directly related to the ORR (oxygen reduction reaction) in the cathode. Catalysts can promote electron transfer and enhance ORR. Platinum-based cathode catalysts have demonstrated an improvement in oxygen reduction [7]. However, the use of this catalyst is limited because of its high cost, instability, and availability [8]. A huge number of catalyst materials have been tested in MFCs, and perovskite materials have garnered great interest recently [9,10,11,12]. The cathode electrode is one of the major governing parameters, as it controls the cathodic reduction reaction, and poor cathode kinetics lower the performance of the MFC.

Perovskite belonging to (ABO_3_)-type oxides is best known for its use as an electrocatalyst due to its structural stability and higher surface area; moreover, the preparation methods of perovskites are variable and simple [13,14], and it is capable of charging and discharging quickly [15,16].

Among the perovskites that have great appeal as cathode catalysts, ferroelectrics offer the ideal properties for improving the proprieties of the microbial fuel cell because of their spontaneous polarization [17,18]. In recent years, a wide number of ferroelectric materials have been investigated, especially in air-cathode MFCs, and have demonstrated encouraging results in terms of power-density generation and COD removal [19,20].

LiTaO_3_ and LiNbO_3_ are two ferroelectric photocatalysts characterized by notable polarization values of 0.5 C/m^2^ and 0.75 C/m^2^, respectively [21]; these materials have been used as photocatalysts for dye degradation [22] and solar energy application [23,24]. LiTaO_3_ and LiNbO_3_ have also been applied as photocatalysts in microbial fuel cells with and without light conditions [25,26]. The results obtained for these two materials are encouraging, achieving a power density of 131 mWm^−3^ for LiNbO_3_ and 55 mWm^−3^ for LiTaO_3_ in light conditions. For the COD removal, LiNbO_3_ and LiTaO_3_ lead to an elimination rate within 66–84%. They have showed that the incorporation of a small ratio of metal ions in the mixed-oxide matrix can control the optical proprieties and the polarization behavior, and several studies have explored the relationship between ferroelectric behavior and catalytic properties by introducing metallic elements with different valences (Ag^+^, Cu^2+^, Mg^2+^, W^6+^) into cathodic components, thereby modulating polarization effects and influencing their optical performance [20,27,28]. From this point of view, the solid solution of these two materials (LiNbO_3_ and LiTaO_3_) doped with Mg (Li_0.95_Ta_0.76_Nb_0.19_Mg_0.15_O_3_) has been investigated in a previous study [29]. The incorporation of 15% of Mg increased the power density of the MFC to 228 (mWm^−2^) under light conditions, and the COD removal to 95.74%. This study aims to investigate the catalytic performance of a new solid solution, LiTa_0.6_Nb_0.4_O_3_, and its Mg doped material Li_0.95_Ta_0.57_Nb_0.38_Mg_0.15_O, as well as evaluating the electrochemical performance. In MFC applications, material performance was further examined by COD removal efficiency and polarization measurements.

## 2. Materials and Methods

### 2.1. Catalysts Elaboration

To synthesize our catalysts, the first step is to elaborate LiNbO_3_ and LiTaO_3_. The precursor materials used for the synthesis are Li_2_CO_3_ (SOLVACHIM, 99%), Ta_2_O_5_ (SigmaAldrich, 99%), and Nb_2_O_5_ (SigmaAldrich, 99.5%); the reagents were mixed stoichiometrically and then ground finely in the presence of ethanol. The mixtures were placed into a muffle furnace at 600 °C for 24 h. The obtained powder was ground again and heated at 800 °C for 48 h.

To obtain LiTa_0.6_Nb_0.4_O_3_, the obtained LiNbO_3_ and LiTaO_3_ was mixed stoichiometrically and heated at 900 °C for 24 h to incorporate Mg in our catalyst, magnesium oxide MgO (99.9%) was weighed and mixed stoichiometrically and then subjected to heat treatments at 1000 °C for 24 h in a muffle furnace to prepare the final products via the ceramic route; the sample was slowly cooled following the heat treatment.

### 2.2. Catalyst Characterization Methods

The phase identification and crystalline structure of the synthesized materials were investigated by XRD analysis, utilizing a Analytical X’pert Pro diffractometer (Malvern Panalytical B.V., Almelo, The Netherlands) at 25 °C, with Cu Kα radiation over a 2theta range from 10° to 70° with a step of 0.06°/s. The elemental analysis and the surface morphology of the synthesized materials were investigated using an environmental Scanning Electron Microscope Energy coupled to Dispersive X-ray (SEM/EDX), FEI Quanta 200 (FEI Company (USA)). The optical properties of the studied sample were collected using a UV–Vis spectrophotometer (Specord 210 Plus, Analytik Jena, Thuringia, Germany), between 200 and 900 nm. The thermic analysis of the obtained materials was carried out with a thermogravimetry-analysis TGA coupled to differential scanning calorimetry (DSC), (METTLER TOLEDO, Barcelona, Spain).

### 2.3. Air-Cathode MFC Configuration and Operation

**Figure 1:**
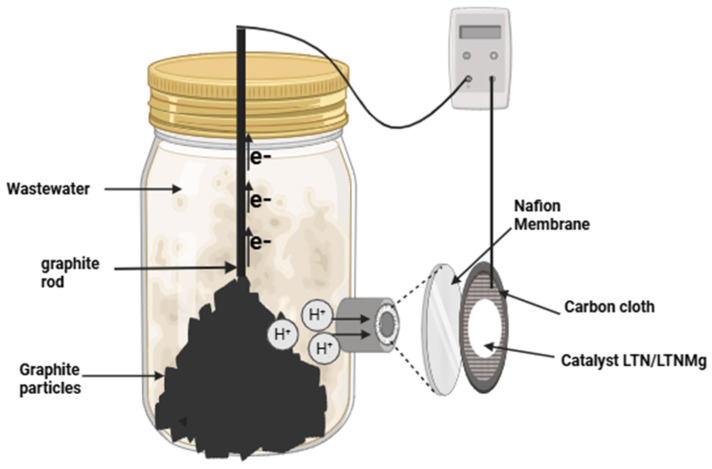
Schematic illustration of air-cathode MFC configuration.

The performance of LTN and LTNMg materials as cathodic catalysts in the MFCs were evaluated using a single-chamber microbial fuel cell. The reactor forming the anodic chamber was a 250 mL glass bottle with a double jacket. The anodic compartment was inoculated with 125 mL of domestic wastewater from ENSAM’s student residence, and was equipped with 100 cm^3^ of graphite particles and a graphite rod (3.2 mm). The air cathode consisted of a carbon cloth coated with the synthesis materials of LTN and LTNMg. To prepare the cathode, 60 mg of each catalyst was mixed with isopropanol, water and PTFE, and then pressed mechanically onto a carbon cloth section measuring 3 cm^2^, loading it to an active section of 1 cm^2^ (Figure 1).

The anodic and cathodic compartments were separated using a proton exchange membrane (PEM) measuring 4 cm in diameter.

Polarization measurement consists of the determination of the generated current of MFC at different external resistances (1Ω–11 MΩ). The polarization curves were obtained from the corresponding voltage data. The open-circuit voltages (OCVs) of air-cathode MFCs were measured using a voltage meter. The current density (I) was given by I = RV external voltage, and the power density (P) was calculated by the following relationship:

$$P=\frac{V^{2}}{R\cdot A}$$

where (V) is the cell voltage, (R) the external resistance, and (A) the effective surface area of the cathode catalyst.

### 2.4. COD Removal 

The COD determination was performed following the standard dichromate method (APHA) [30]. The analysis was conducted using a photoLab 7600 UV–VIS spectrophotometer (WTW GmbH, Xylem Analytics, Weilheim, Germany). COD removal efficiency was determined using the following (Equation (1)):

(1) $$CODRemoval(\%)=\frac{CODi-CODf}{CODi}\times 100$$

CODi and CODf are the initial and final COD concentrations (mg/L) of the effluent in the anodic chamber, at a given time.

### 2.5. Electrochemical Measurements

A PGZ 100 potentiostat was used for the electrochemical analysis of the anode. Carbon cloth electrodes with a coated surface area of 2 cm^2^ served as the working electrodes, while the counter electrode was a platinum grid. All potentials were controlled using a conventional three-electrode system with a saturated calomel reference electrode. Polarizations were performed at −0.2 V s. SCE, E0 = 0.241 V vs. SHE, as this potential corresponds to the most negative value capable of producing the maximum current density [31]. All electrochemical experiments were carried out in hermetically sealed containers, each holding 250 mL of domestic wastewater, under ambient room-temperature conditions.

## 3. Results

### 3.1. Catalyst Characterization

**Figure 2:**
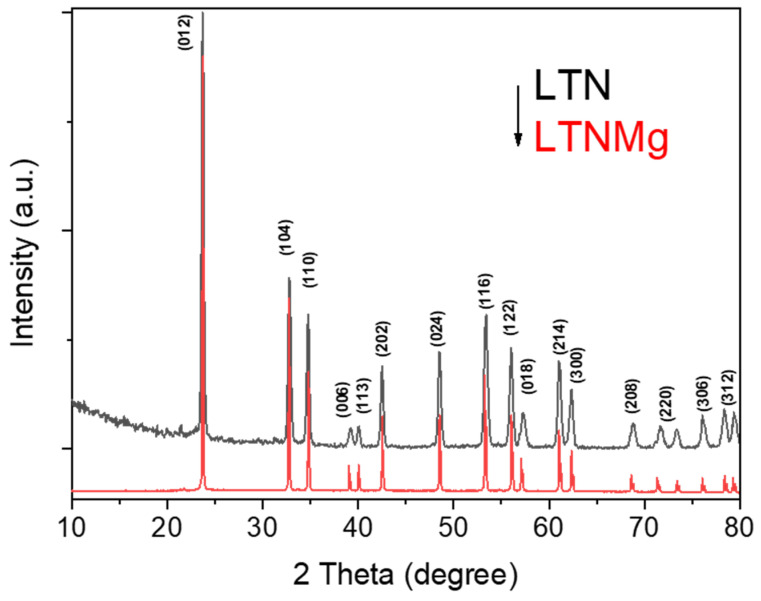
XRD patterns for the investigated LTN and LTNMg materials.

An investigation into the crystal structures of LTN and LTNMg was undertaken using X-ray diffraction (XRD), employing Cu-Kα radiation (λ = 1.549 Å) in the 2θ range from 10° to 80° on a PANalytical X’Pert PRO diffractometer. The results presented show the XRD patterns (Figure 2) of these as-synthesized photocatalysts.

The diffraction pattern of the pure phase corresponds to standard lithium niobate (JCPDS No. 020-0631), characterized by trigonal symmetry and space group R3c. Sharp and well-defined diffraction peaks indicate the high crystallinity and structural ordering of the synthesized samples. Interestingly, the XRD patterns showed all samples had preserved the trigonal structure with no new peaks, indicating that the incorporation of Mg does not affect the lithium niobate crystal structure.

**Figure 3:**
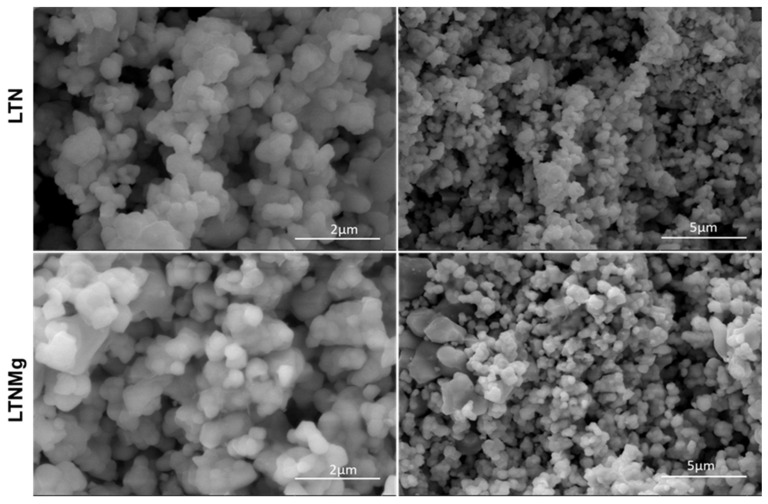
SEM images of the synthetized LTN and LTNMg.

**Figure 4:**
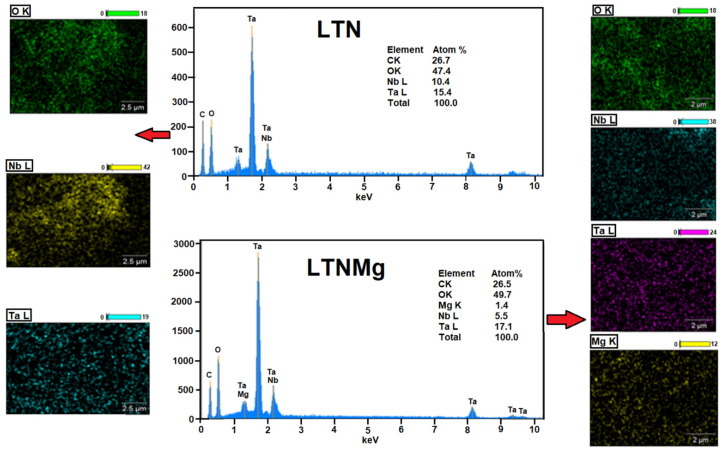
Mapping–EDX of LTN; mapping-EDX of LTNMg.

Figure 3 presents the detailed characterization by scanning electron microscopy (SEM) of the LTN and LTNMg catalysts before their use in the MFC device. The SEM images of the catalysis show a homogeneous distribution of grains, with a spherical morphology for all the compositions (Figure 4). However, a difference in size is observed between LTN and LTNMg, with the latter exhibiting larger dimensions than LTN. X-ray energy-dispersive spectroscopy analysis (EDX) was used to determine the composition of the materials. The pattern presented in Figure 4 confirms the presence of the expected elements, namely (Ta, Nb and O) for LTN, and (Ta, Nb, O and Mg) for LTNM.

This homogeneous distribution of elements was confirmed by mapping–EDX analysis; the absence of Li is explained by its low atomic mass, which makes it difficult to detect by EDX. In addition, the presence of carbon in the EDX spectrum results from the material used as a support during SEM analysis.

**Figure 5:**
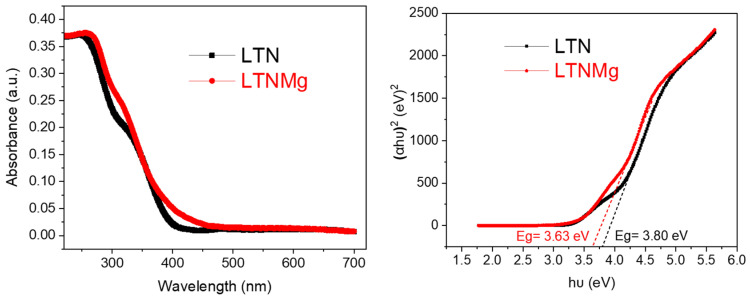
UV–VIS spectroscopy and Tauc Plot’s for LTN and LTNMg.

The UV–Vis absorption spectra (Figure 5) of the synthesized photocatalysts were examined to explore their optical properties. The analysis revealed that the samples predominantly absorb light in the ultraviolet region. However, the LTN catalyst exhibited slightly lower UV absorption intensity (254 nm) compared to the doped counterpart, LTNMg (265 nm). This observation suggests that the introduction of dopants into the host lattice enhances light absorption, which could be beneficial for photocatalytic applications.

To further evaluate the optical properties, the optical bandgaps of LTN and LTNMg were determined using Tauc’s equation (Equation (2)). The estimated bandgap values were approximately 3.8 eV for LTN and 3.63 eV for LTNMg, as illustrated in the inset of Figure.

(2) αhν=A(hν−Eg)n2

In microbial fuel cells, ORR efficiency depends on how effectively electrons can be transferred to O_2_. The Tauc plot indicates a band gap of 3.8 eV for LTN and 3.63 eV for LTNMg. By shifting from 3.8 eV (corresponding to 326 nm) to 3.63 eV (corresponding to 342 nm), the material becomes slightly more sensitive to near-UV, which increases the amount of absorbed light and thus potentially generates more electron–hole pairs, improving charge transfer to adsorbed oxygen molecules at the cathode.

### 3.2. Electrochemical Performance

To evaluate the electrochemical performance of the catalysts, we compared their electrocatalytic activities to those of the conventional catalyst commonly used in microbial fuel cells for the oxygen reduction reaction [32].

#### 3.2.1. Cyclic Voltammetry

Cyclic voltammetry was used to study the electrochemical behavior of non-modified carbon cloth and catalyst-coated carbon cloth electrodes with LiTa_0.6_Nb_0.4_O_3_ (LTN) and Li_0.95_Ta_0.57_Nb_0.38_Mg_0.15_O_3_ (LTNMg) for the oxygen reduction reaction (ORR). The experiments were conducted in an oxygen-saturated wastewater solution, sweeping the potential between −1 V and 1 V vs. Ag/AgCl at a scan rate of 50 mV/s.

**Figure 6:**
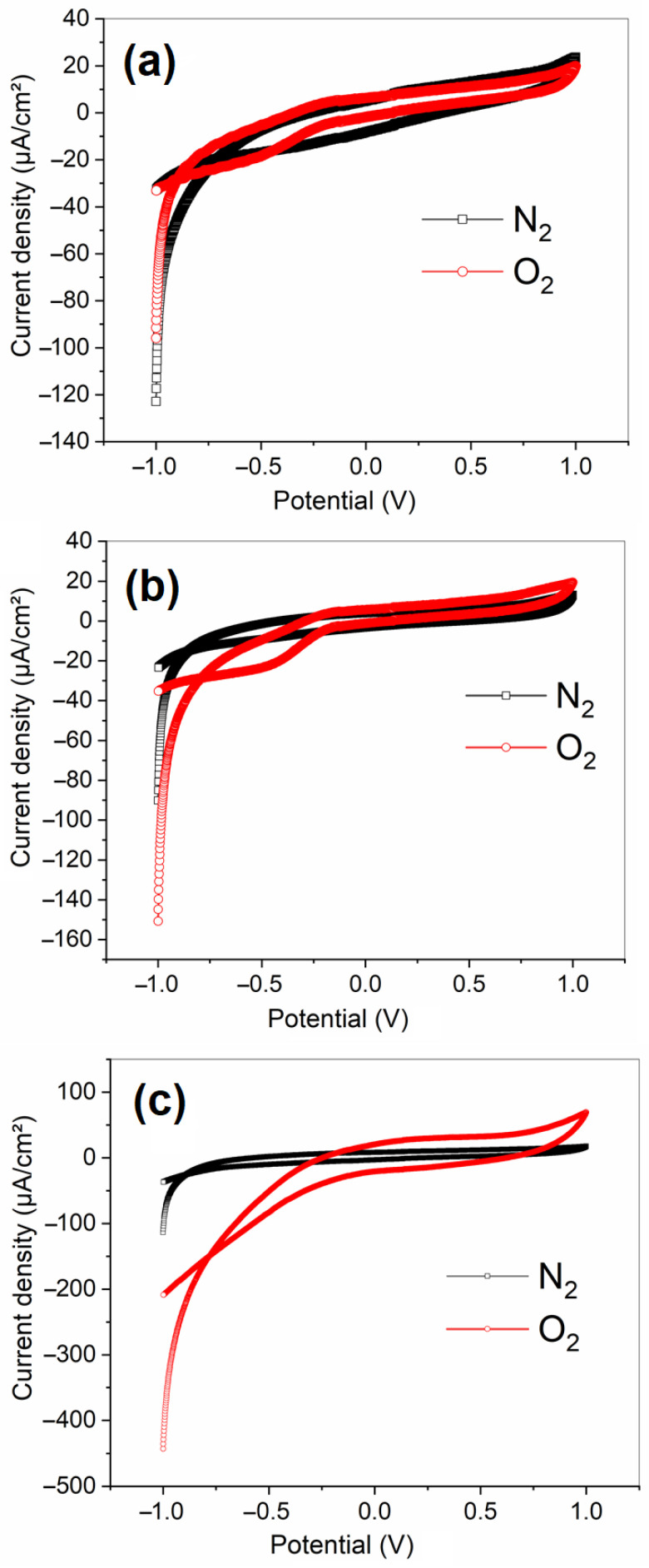
Cyclic voltammetry curve of (**a**) untreated carbon cloth electrodes in an O_2_-saturated solution; (**b**) LTN-coated carbon cloth electrodes in an O_2_-saturated solution; (**c**) LTNMg-coated carbon cloth electrodes in an O_2_-saturated solution.

Figure 6b shows the CVs for carbon cloth electrode modified with LiTa_0,6_Nb_0,4_O_3_ (LTN). In the O_2_-saturated solution, well-defined peaks are observed, attributed to oxygen reduction (ORR). This indicates that LTN acts as an electrocatalyst for ORR. Conversely, in an N_2_-saturated solution, the absence of peaks confirms that the peaks seen in oxygenated media are indeed due to O_2_ reduction.

Figure 6c shows the CVs for carbon cloth modified with Li_0.95_Ta_0.57_Nb_0.38_Mg_0.15_O_3_ (LTNMg). The oxygen reduction peak is more intense than for LTN, indicating a higher current density and a better catalytic performance towards ORR. This suggests that the addition of Mg in LTN enhances its electrocatalytic activity for O_2_ reduction, making LTNMg a more effective catalyst for this reaction than LTN.

The CVs highlight the superior ORR activity of LTN- and LTNMg-coated electrodes compared to untreated carbon cloth (Figure 6a), with well-defined reduction peaks in O_2_ media indicating improved ORR kinetics. The onset reduction potentials are higher for LTN (−0.15 V vs. Ag/AgCl) and LTNMg (−0.10 V) compared to untreated carbon cloth (−0.25 V). The higher catalytic activity for LTNMg can be attributed to the presence of Mg creating structural defects that favor O_2_ adsorption, the reduced optical gap facilitating electron transfer, and the synergy between the active sites of Nb and Mg that can catalyze the O-O bond cleavage [24,28].

#### 3.2.2. Linear Sweep Voltammetry (LSV)

**Figure 7:**
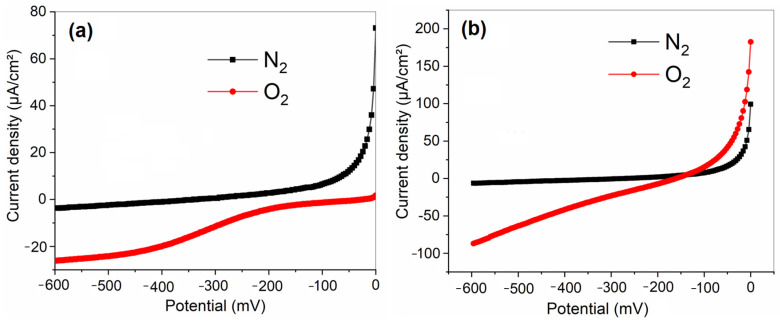
LSV of the LTNMg-modified carbon cloth electrode in a (**a**) wastewater solution saturated with O_2_ and N_2_, at a scan speed of 50 mV/s; (**b**) wastewater solution saturated with O_2_ and N_2_, at a scan speed of 50 mV/s.

The analysis of linear sweep voltammetry curves for carbon cloth electrodes modified with LTN and LTNMg in a wastewater solution saturated with oxygen and nitrogen (Figure 7) reveals significant differences in their electrocatalytic performance for the oxygen reduction reaction (ORR). The electrode modified with LTN shows a well-defined cathodic peak around −0.5 V vs. Ag/AgCl, with a maximum cathodic current density of about −1.2 mA/cm^2^, indicating moderate activity for ORR. The relatively narrow peak shape suggests relatively fast electron transfer kinetics during the electrochemical reduction of oxygen. In contrast, the electrode modified with LTNMg shows a cathodic peak shifted to slightly more negative potentials, around −0.6 V vs. Ag/AgCl, but with a significantly higher cathodic current density, reaching about −2.2 mA/cm^2^. This increased current density indicates better electrocatalytic activity for ORR compared to the electrode modified with LTN. However, the broader cathodic peak observed for LTNMg may indicate diffusion limitations or slower electron transfer kinetics during oxygen reduction on this modified electrode.

#### 3.2.3. Chronoamperometry

**Figure 8:**
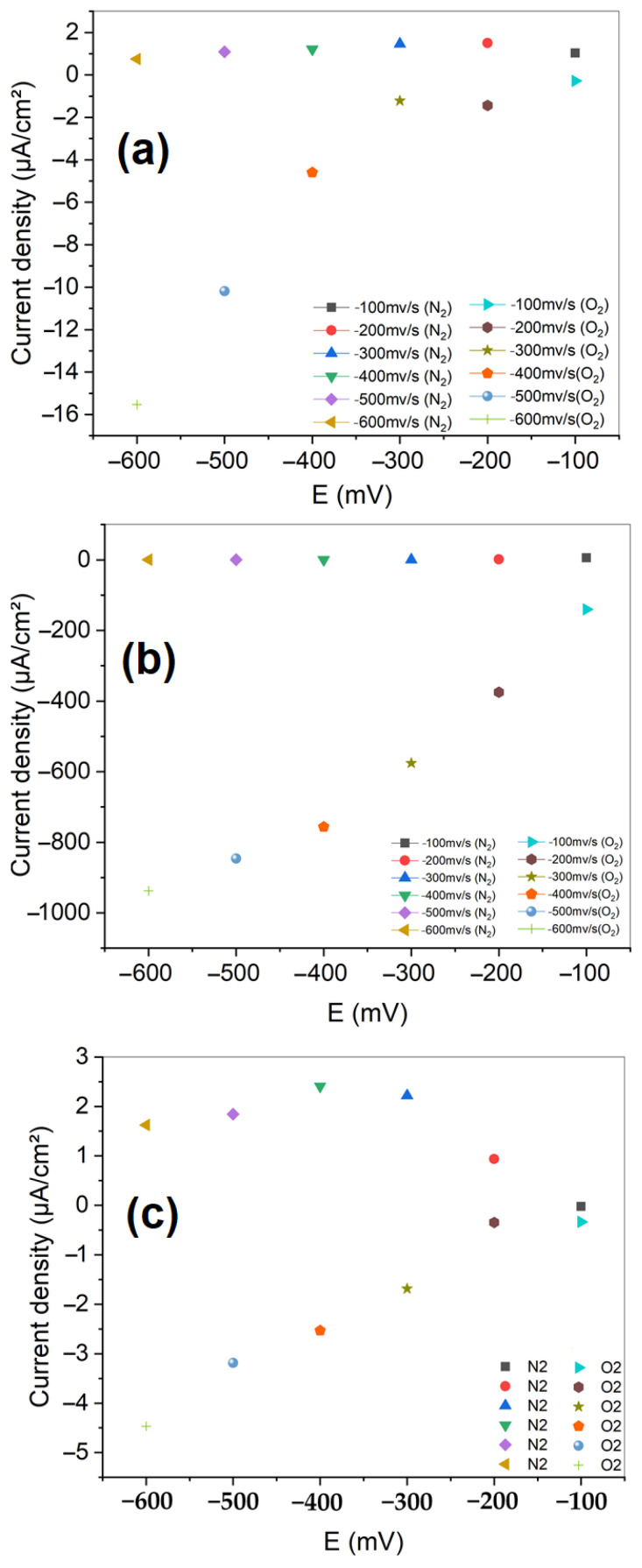
Chronoamperometry of (**a**) untreated carbon cloth electrode; (**b**) LTN-coated carbon cloth electrode; and (**c**) LTNMg-coated carbon cloth electrode.

The analysis of chronoamperometric curves (Figure 8) for two electrodes in an oxygen and nitrogen-saturated medium provides valuable insights into their performance and stability for the oxygen reduction reaction (ORR). The untreated carbon cloth electrode shows a relatively low initial cathodic current, around −0.2 mA/cm^2^, which gradually decreases over time, indicating low initial activity and probable deactivation due to surface instability under these conditions.

In contrast, the electrode coated with LTN shows a significant improvement with a much higher initial cathodic current, around −1.2 mA/cm^2^, indicating increased ORR activity due to surface modification. However, this electrode also experiences a significant decrease in cathodic current over time, reaching about −0.6 mA/cm^2^, suggesting a potential loss of activity related to poisoning or degradation of the modified surface. Among the three electrodes studied, the carbon cloth electrode coated with LTNMg exhibits the best initial performance and the greatest stability for ORR in this medium. Its initial cathodic current is the highest, exceeding −2.5 mA/cm^2^, and although it undergoes a slight decrease, it stabilizes around −2.2 mA/cm^2^, indicating the better durability of this modified electrode compared to the other two. This relative stability could be attributed to greater resistance to poisoning or surface degradation phenomena under these experimental conditions.

#### 3.2.4. Electrochemical Impedance Spectroscopy EIS

Electrochemical Impedance Spectroscopy (EIS) is a powerful method for examining in detail the electrochemical behavior of systems. In the context of microbial fuel cells (MFC), this technique allows us to quantify the contribution of different internal resistances to the total resistance of the device.

**Figure 9:**
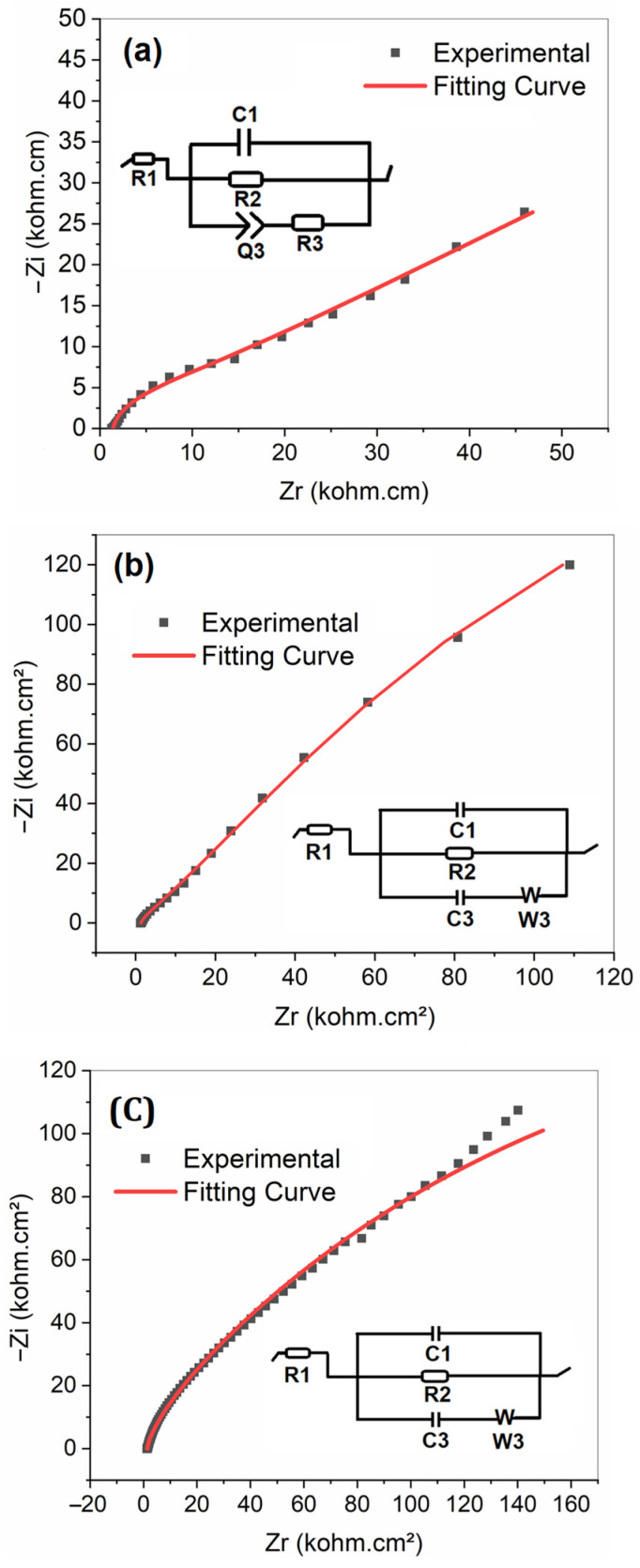
Electrochemical impedance spectroscopy of (**a**) untreated carbon cloth; (**b**) LTN-coated carbon cloth; and (**c**) LTNMg-coated carbon cloth.

Nyquist plots were established to compare the performance of untreated carbon cloth cathode, and the modified one by LTN and LTNMg, within the MFC system. The analysis of these graphs was carried out using equivalent circuits, modeled via the EC-Lab software, version V11.41 (Figure 9).

The analysis of Nyquist plots and equivalent circuits for the three types of cathodes reveals significant differences in their electrochemical properties. Unmodified carbon cloth exhibits an ohmic resistance of 1.465 Ohms and an extremely high charge transfer resistance, indicating low electrical conductivity and very high charge transfer resistance. This suggests limited performance as a cathode in an MFC system.

The coating with LTN significantly improves the electrochemical properties. The ohmic resistance slightly decreases to 1.359 Ohms, while the charge transfer resistance drastically drops to 459.5 Ohms. There is also an increase in double-layer capacitance (2.875 × 10^−3^ F) and the introduction of diffusion processes, represented by a Warburg element (W = 20.33 Ohm.s^−1/2^). These changes indicate a significant improvement in conductivity and a reduction in charge transfer resistance, likely due to the crystalline structure and electronic properties of LTN.

Modification with LTNMg brings additional changes. The ohmic resistance (1.441 Ohms) remains lower than that of unmodified carbon cloth. The double-layer capacitance further increases (3.751 × 10^−3^ F), and the charge transfer resistance (588.1 Ohms) is slightly higher than for LTN but still much lower than that of carbon cloth. The most notable change is the increase in charge storage capacity (C3 = 773,164 F) and the intensification of diffusion processes (W = 43.1 Ohm.s^−1/2^). These improvements can be attributed to the presence of magnesium in the structure, which influences the electronic properties and surface reactivity of the material. These results show a clear progression in the improvement of electrochemical properties: carbon cloth alone exhibits the lowest performance, the addition of LTN significantly enhances conductivity and reduces charge transfer resistance, while LTNMg further optimizes charge storage capacity and diffusion processes. Although LTNMg shows a slight increase in charge transfer resistance compared to LTN, its advantages in terms of storage capacity and diffusion may offset this minor drawback. These surface modifications with complex oxides of lithium, tantalum, niobium, and magnesium thus appear promising for improving the performance of cathodes in microbial fuel cell systems, mainly by increasing charge storage capacity and facilitating diffusion processes. The unique properties of these materials, particularly their crystalline structure and chemical composition, likely play a crucial role in the observed improvements in electrochemical performance [33,34].

### 3.3. Catalysts Power Performance and Wastewater Treatment in Air-Cathode MFCs

#### 3.3.1. Power Performance

**Figure 10:**
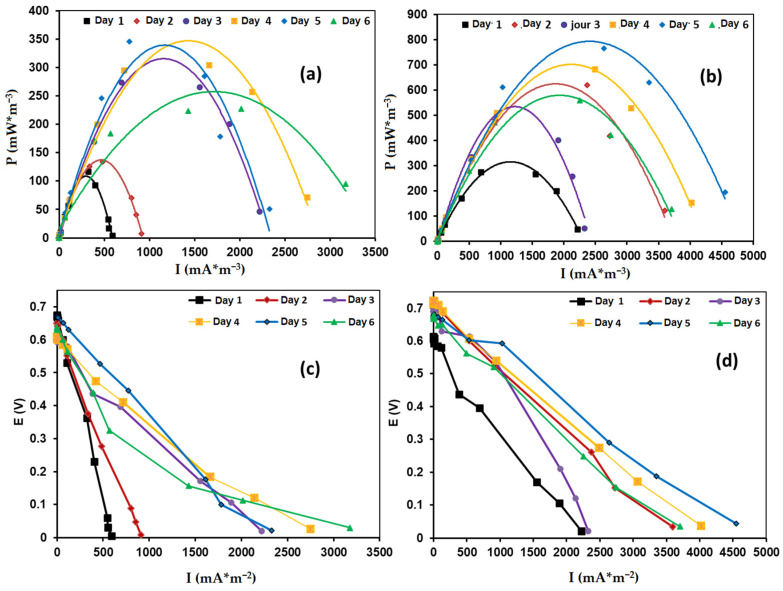
Power-density curves of the air-cathode MFC (**a**), LTN, (**b**) and LTNMg, and the polarization curves of (**c**) LTN and (**d**) LTNMg.

Figure 10a,b present power-density curves over six days of MFC operation, comparing LTN (Figure 10a) and Mg-doped LTN (LTNMg, Figure 10b). The Mg-doped catalyst achieved the highest power output, ranging from 380 to 764 mW·m^−2^, with peak performance recorded on day 5. In contrast, undoped LTN exhibited power outputs between 104 and 410 mW·m^−2^, varying with operational day. Notably, the incorporation of Mg^2+^ into the LTN structure nearly doubled the power density. This enhancement is likely due to improved charge transfer facilitated by the Mg^2+^-induced modification of the LTN crystal lattice, leading to pronounced interfacial polarization. These findings are consistent with the electrochemical performance observed for both catalysts. The polarization data presented in Figure 10c,d were collected by applying various external resistance loads ranging from 1 Ω to 11 MΩ. The characteristic curves indicate two inflection points that mark three distinct operational phases normally observed in MFC devices. The first phase is due to the rapid voltage drop caused by activation losses at high external resistance [35,36,37]. The second phase is characterized by a linear voltage drop, reflecting the system’s ohmic losses [38]. The slope of this section is particularly informative; a steeper slope indicates higher internal resistance, correlating with the challenges in charge transfer and resistance within the cell components. The final phase, defined by a second rapid voltage drop at high current densities, corresponds to mass transport limitations, likely exacerbated by the intrinsic properties of the cathodes and system design. These insights from the polarization curves complement the previously mentioned fundamental loss areas—activation, ohmic, and charge losses—and further elaborate on how internal resistance impacts the cell’s overall performance, particularly in power production.

#### 3.3.2. Wastewater Treatment

The wastewater treatment performance of the MFC was evaluated by measuring the COD removal rates. Table 1 illustrates wastewater proprieties before treatment in MFC’s device.

**Figure 11:**
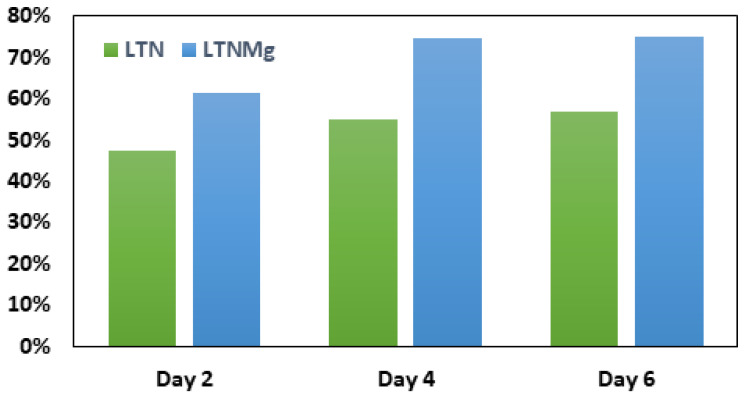
COD removal over days in MFC with LTN and Mg-doped LTN cathode catalysts.

By comparing the initial and final COD levels before passing through the MFC system and after six days of operation (Figure 11), the effectiveness of COD removal was assessed for the two MFCS with LTN and LTNMg cathode catalysts. Within just two days of operation, the MFC incorporating LTNMg achieved a COD elimination percentage of 61.2%, and the MFC with non-doped LTN reached a 47.5% removal rate of COD. After six days of MFC operation, the COD abatement reached its peak for both MFCs with different catalysts, achieving a rate of 75% for LTNMg, while it did not exceed 57% for LTN. The results obtained for COD removal are in good agreement with the power densities reached for each solid solution.

Table 2 highlights a performance comparison between our solid solution LTNMg and the same solid solution with a different stochiometric coefficient [29]. Li_0.95_Ta_0.57_Nb_0.38_Mg_0.15_O_3_ (764 mW m^−2^) shows a much higher maximum power density than Li_0.95_Ta_0.76_Nb_0.19_Mg_0.15_O_3_ (228 mW m^−2^), making it more efficient for energy applications. This significant difference in power densities can partly be attributed to the band gap. In fact, the wider band gap characteristic to Li_0.95_Ta_0.76_Nb_0.19_Mg_0.15_O_3_ at 4.03 eV generally limits the flow of the electrical current and reduces the material’s electrical conductivity, leading to lower power densities. The initial COD can also influence its power performance; a higher initial COD means more substrate for the microorganisms to metabolize, which can lead to increased electron production and, consequently, higher power output [39].

## 4. Conclusions

This comprehensive study on the ferroelectric materials LiTa_0.6_Nb_0.4_O_3_ (LTN) and Li_0.95_Ta_0.57_Nb_0.38_Mg_0.15_O_3_ (LTNMg) as cathodic catalysts in MFCs revealed promising results. The ceramic synthesis produced high-purity materials, confirmed by XRD, showing a well-defined crystalline structure isotype to LiTaO_3_. Optical properties reveal band-gap energies of 3.63 eV for LTNMg and 3.8 eV for LTN. Morphological and compositional examinations by SEM-EDX confirmed the expected elemental composition and revealed a homogeneous granular structure.

Electrochemical performance highlighted the remarkable efficiency of LTNMg compared to LTN and unmodified carbon cloth. Cyclic and linear voltammetry showed better catalytic activity of LTNMg for the oxygen reduction reaction (ORR), with higher cathodic current density (−2.2 mA/cm^2^). Chronoamperometry confirmed the superior stability of LTNMg over time. Electrochemical impedance spectroscopy revealed significant improvements in electrochemical properties for both materials, with LTNMg showing high charge storage capacity and enhanced diffusion processes.

The incorporation of magnesium into the LTN structure to form LTNMg led to significant improvements in electrocatalytic properties for ORR, attributable to the creation of structural defects promoting oxygen adsorption, a reduction in the optical gap facilitating electron transfer, and potential synergy between Nb and Mg active sites catalyzing the O-O bond breakage. These results suggest that LTNMg represents a promising catalyst for MFC cathodes, offering a potential alternative to conventional material.

Electrochemical results were confirmed by testing LTNMg and LTN as cathode catalysts in an air-cathode MFC. In terms of electricity production and wastewater purification, the Li_0.95_Ta_0.57_Nb_0.38_Mg_0.15_O_3_ (LTNMg) ferroelectric cathode catalyst demonstrated impressive results in the MFC device performance. After 6 days of operation, it achieved a high COD removal rate of approximately 75%, allowing for the generation of a maximum power density of 764 mWm^−2^.

## Figures and Tables

**Table 1:** Wastewater properties before treatment in MFC device.

Provenance	COD	DO	Temperature	Conductivity	pH
Urbain waste water	864 mg/L	6.04	25 °C	1.99 mS	7.72

**Table 2:** Comparison of LTNMg catalyst efficiencies in MFC.

Solid Solution	Band Gap (eV)	COD Initial (mgL^−1^)	OCV (mV)	Maximum Power Density (mWm^−2^)	COD Removal (%)	References
Li_0.95_Ta_0.57_Nb_0.38_Mg_0.15_O_3_	3.60	864	680	764	75	Present study
Li_0.95_Ta_0.76_Nb_0.19_Mg_0.15_O_3_	4.03	471	460	228	95.7	[29]

## Data Availability

Data are contained within the article.
